# The impact of verbal encouragement during the repeated agility speed training on internal intensity, mood state, and physical enjoyment in youth soccer players

**DOI:** 10.3389/fpsyg.2023.1180985

**Published:** 2023-09-21

**Authors:** Okba Selmi, Hilmi Jelleli, Souheir Bouali, Bilel Aydi, Omar Hindawi, Antonella Muscella, Anissa Bouassida, Katja Weiss, Beat Knechtle

**Affiliations:** ^1^High Institute of Sports and Physical Education of Kef, University of Jendouba, El Kef, Tunisia; ^2^Research Unit: Sportive Sciences, Health and Movement, El Kef, Tunisia; ^3^Laboratory of Analysis and Expertise of Sports Performance, Institute of Science and Technology of Physical and Sports Activities, Abdelhamid Mehri, University Constantine, Constantine, Algeria; ^4^Department of Sports Rehabilitation, The Hashemite University, Zarqa, Jordan; ^5^Department of Biological and Environmental Science and Technologies (DiSTeBA), University of Salento, Lecce, Italy; ^6^Institute of Primary Care, University of Zurich, Zürich, Switzerland; ^7^Medbase St. Gallen Am Vadianplatz, St. Gallen, Switzerland

**Keywords:** verbal encouragement, repeated agility, motivation, enjoyment, mood

## Abstract

**Objective:**

Verbal encouragement (VE) can be used by coaches to boost morale and commitment during training exercises. This investigation aimed to study the impacts of VE given by coaches on the physiological aspects, players' internal intensity, mood, and perceived enjoyment of youth soccer players during repeated agility speed training (RAS).

**Methods:**

A total of 17 male youth soccer players (mean ± SD; age: 13.8 ± 0.4 years; body mass: 59.1 ± 6.7 kg; height: 170.0 ± 6.2 cm; training experience: 5.1 ± 0.7 years) participated, in a randomized order, in two experimental training sessions that consisted of a RAS (i.e., the Illinois course) either with VE (RAS-E) or without VE (RAS-NE), with a 7-day interval between the testing sessions. Heart rate (HR) was registered throughout the exercise. The rating of perceived exertion, blood lactate concentration [La], and perceived enjoyment were measured after each training session. The mood state was recorded before and after each protocol.

**Results:**

HR mean (Cohen's coefficient *d* = 0.45, small), %HRmax (*d* = 0.37, small), HR peak (*d* = 0.66, moderate), [La] (*d* = 0.56, small), and the PACES score (*d* = 2.8, very large) were higher in RAS-E compared to RAS-NE (all, *P* < 0.001). Compared to the RAS-E trial, the RAS-NE trial showed higher fatigue (*P* < 0.01), tension (*P* < 0.05), anger (0.05), total mood score (*P* < 0.001), and lower vigor (*P* < 0.001).

**Conclusion:**

Coaches may use VE during RAS to improve psychophysiological responses, mood state, and perceived enjoyment in youth soccer players.

## Introduction

To increase soccer players' performance, both physical and psychological components must be developed, and young soccer players must be inspired (Nobari et al., 2021; Ouertatani et al., 2022). Among soccer's most important physical aspects are the repeated intense efforts that require significant physical fitness (Chmura et al., 2022; Marzouki et al., 2022). For example, repeated sprint ability is a very interesting physical quality in soccer (Walklate et al., 2009; Haj-Sassi et al., 2011; Fessi et al., 2016; Hammami et al., 2017). Most coaches use repeated agility speed (RAS) training to increase soccer players' physical fitness (Walklate et al., 2009). Recent studies have reported that in soccer, physical capacity can be developed or maintained through specific training exercises, including RAS conditioning performed at smaller distances (Haj-Sassi et al., 2011; Fessi et al., 2016). It has recently been proposed that repeated efforts must be encouraged for physical and affective improvement and could be more effective than other training methods in improving positive emotional responses (Sahli et al., 2022).

During soccer training, youth athletes increase their physical skills as well as their cognitive and emotional capacities, but continual stimuli offered by coaches and physical coaches are required to generate active involvement and positive feelings (Selmi et al., 2017a). This stimulation may be generated verbally, promoting more motivation and higher engagement during training (Sahli et al., 2022). This, in turn, improves the desire for physical exercise and positive feelings (Kilit et al., 2019). It has been shown that coaches' verbal encouragement (VE) increases the physical and physiological demands of physical activity, especially repeated efforts (Selmi et al., 2017a; Kilit et al., 2019; Hammami et al., 2021; Sahli et al., 2022). A coach's or a physical coach's VE is seen as an external incentive that favorably affects concentration, physical commitment, positive emotions, and the desire to exercise (Selmi et al., 2017b).

Moreover, the motivation that comes from training exercises may be linked to a positive mood state, physical enjoyment, and an elevation of exercise intensity (Neto et al., 2015; Edwards et al., 2018). Several scientific researchers have confirmed the effect of VE on internal intensity (Rampinini et al., 2007; Selmi et al., 2017b; Sahli et al., 2020). For example, Rampinini et al. reported that in four variants of short-sided games (SSG) (3-, 4-, 5-, and 6-a-side), the values of lactate concentration [La], heart rate (HR), and rating of perceived exertion (RPE) during SSG with VE were significantly higher than those of SSGs without VE (Rampinini et al., 2007). Sahli et al. (2020) stated that VE from an expert translates into favorable motivation, enjoyment, and mood state, thereby improving physiological demands during SSG in adolescent soccer players. Additionally, Aydi et al. (2022) indicated that encouragement cues from practitioners ensure higher physiological responses, physical performance, a positive mood, and physical enjoyment during a ball-training circuit (Hoff circuit) in adolescent players. Little is known regarding soccer training in terms of how VE affects the physical and physiological aspects. To the best of our knowledge, no study has addressed the effects of VE on physiological responses, internal intensity, and psychological aspects during RAS training in young soccer players.

Accordingly, this investigation aimed to determine the impact of VE given by coaches on physiological responses, players' internal intensity, and emotional responses during RAS training in youth soccer players. We hypothesized that RAS-E would produce higher exercise intensity, physical pleasure, and positive mood than RAS-NE.

## Materials and methods

### Participants

A total of 17 male youth soccer players participated in the investigation (mean ± SD: age: 13.8 ± 0.4 years; body mass: 59.1 ± 6.7 kg; height: 170.0 ± 6.2 cm; training experience: 5.3 ± 0.7 years). The inclusion criteria were as follows: (i) all players competed for the same team; (ii) all children had no reported history of illnesses or injuries 8 weeks before and during the experimental period; (iii) no physical or cognitive disease was reported; and (iv) regular presence of participants in training sessions.

Players and their parents agreed to take part in the investigation and gave recorded informed written approval after clarification about the requirements, risks, and benefits associated with participation.

### Procedures

**Figure 1:**
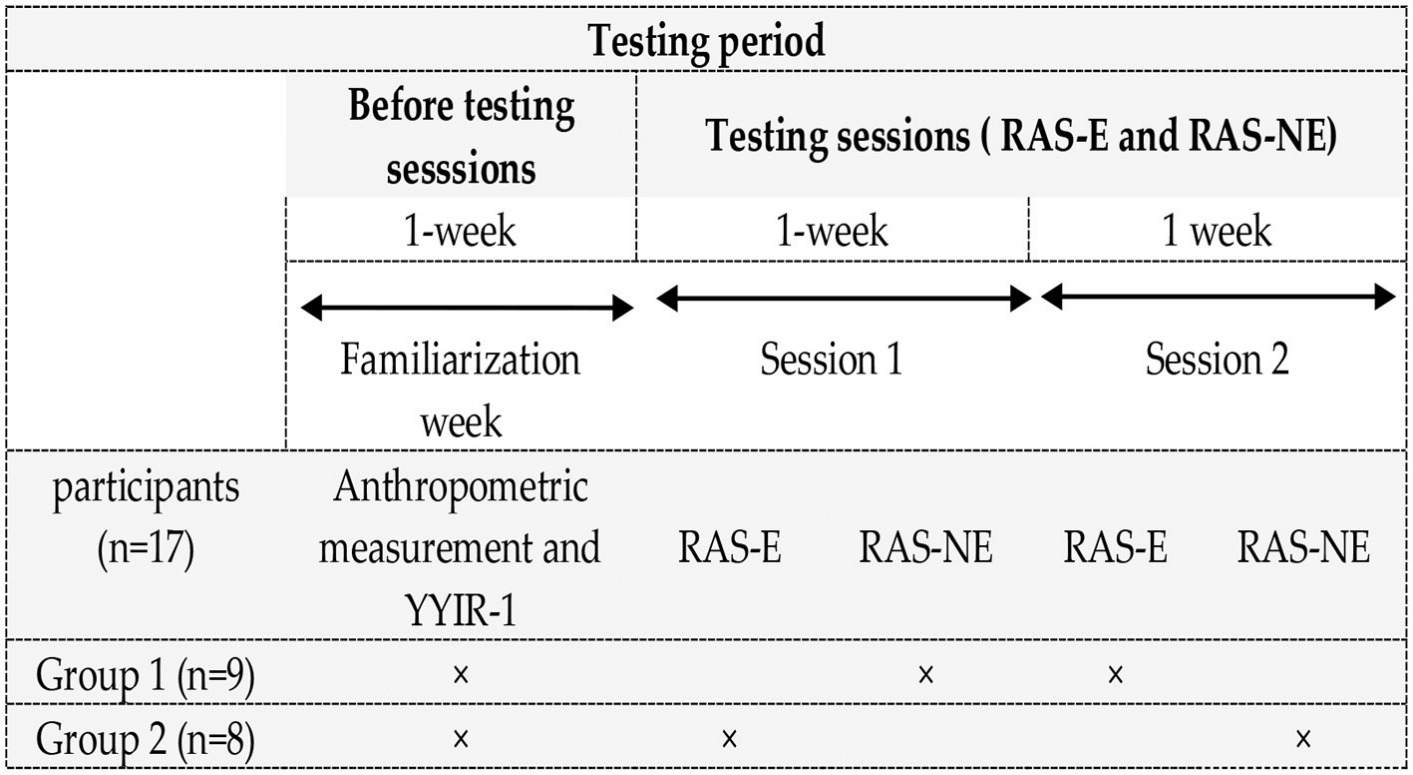
Diagram of the test's procedures. RAS-E, repeated agility speed with VE; RAS-NE, repeated agility speed without VE.

The investigation was carried out during the 2022–2023 competitive soccer season. Before the beginning of the experimental study, height and body weight were assessed, and the maximal heart rate (HRmax) of the children was determined through the Yo-Yo intermittent recovery test level-1 (YYIR-1). In the experimental period, two sessions of RAS training were performed. The sessions were separated by an interval of 7 days. Each protocol [RAS with the coach's VB (RAS-E) and RAS without VE (RAS-NE)] was performed once. During each trial (i.e., RAS-E and RAS-NE), the children were split into two groups, with eight children completing RAS-E and the other nine completing RAS-NE in a randomized order. In total, each child completed the RAS-E and the RAS-NE once (Figure 1). All tests were carried out on the same soccer field and at the same time of day (between 4.00 p.m. and 5.30 p.m.) in order to limit the potential effects of circadian change.

During the RAS training, HR was continuously monitored. RPE, [La], and the perceived enjoyment (PACES) score were assessed after the RAS training. Furthermore, the profile of mood state (POMS) was measured before and after each test (i.e., RAS-e and RAS-NE).

All children abstained from intense exertion for at least 48 h before the experimental tests. Each RAS session was preceded by a standardized warm-up (20 min) involving jogging, proprioception exercises, dynamic stretching, and coordination movements that ended with four 12 m accelerations. Three minutes of passive recovery were given before the RAS exercise. Children were familiarized with the POMS questionnaire, the OMNI-RPE scale, the physical enjoyment scale, and the RAS protocol prior to the investigation.

### The repeated agility speed (Illinois course)

**Figure 2:**
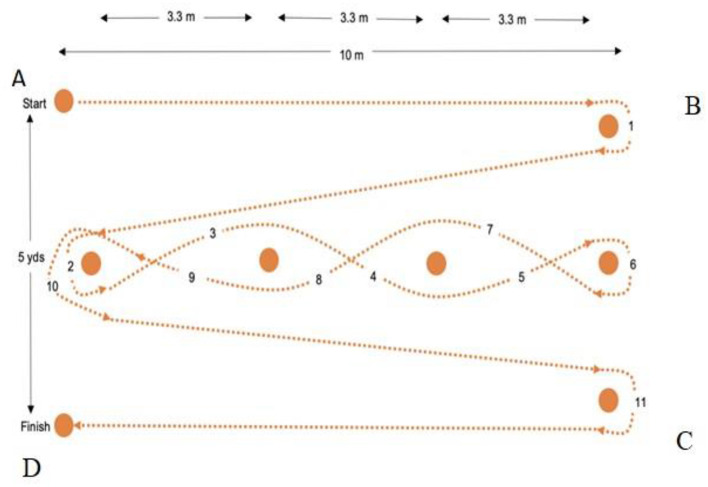
Illustration of the Illinois course exercise. The children dribble the ball through the course. The course is constituted with 10 m long × 5 m wide; cone at point A to determine the start; cone at B and C to determine the turning spots; cone at point D to determine the finish; and the 4 cones are placed in the center of the testing area 3.3 m apart. The children start standing just behind the starting line with the ball in front of their feet; they run the course in the set path (left to right to left); and each trial (one repetition) is complete when they cross the finish line.

The Illinois course was performed in an outdoor field, with the track distance strictly implemented as seen in other studies (Amiri-Khorasani et al., 2010; BenOunis et al., 2013). Participants moved a ball across the course by dribbling. Children were required to perform backward dribbling between cones with maximum speed (Figure 2). The children covered the course as quickly as possible. After each repetition, participants passively recovered for 20 s and then made the next repetition in the opposite direction. The Illinois course was repeated for two bouts of eight repetitions with 3 min of passive recuperation between bouts. The children were asked to make maximum effort throughout the exercise and to do the minimum time possible for each agility speed.

The children performed the RAS-E trial with the coach's VE, while they carried out the RAS-NE trial without VE. The coach stood near the finish line of the course to encourage the children (words of encouragement such as “Go! Go! Go!,” “Again!,” “Faster!,” “More dynamic!,” “More exertion!,” “Courage!,” and “resist”) (Aydi et al., 2022). During the RAS-NE trial, the coach moved next to the course and monitored the children but did not use VE.

### The OMNI-child perceived exertion scales (OMNI-RPE)

The children's OMNI-RPE (0–10) scale (Lagally, 2013) was used immediately upon completion of each intervention (i.e., RAS-E and RAS-NE) to determine the internal intensity of children. This tool was assessed using the question “How was the exercise and how did you feel?” The OMNI scale ranges from 0 to 10 [between Extremely Easy (0) and Extremely Hard (10)] with descriptive terms in combination with images to measure RPE during exercise training. Image aids were used to streamline the understanding of related numerical RPE values. This perceived scale has been validated and used in other scientific research (Lagally et al., 2016; Erichsen et al., 2017).

### Physical activity enjoyment

To assess positive feelings, the PACES (8 items) was used (Lagally et al., 2016) after each RAS intervention (5 min). The physical enjoyment was assessed using a question “How did you feel at the moment of the exercise?” Each item of the PACES is scored on a 7-point scale ranging from 1 (it is very fun) to 7 (it is not pleasant). The PACES score ranges from 8 through 56. Higher enjoyment scores indicated higher levels of perceived enjoyment. In this investigation, Cronbach's α of the perceived physical enjoyment was 0.89.

### Profile of mood states (POMS)

To monitor variation in mood states during training exercises, a POMS questionnaire (24 items) (Mullen et al., 2011) was administered. The tool was given to the children before and after each RAS intervention (i.e., 10 min) to evaluate six sentimental states (vigor, fatigue, depression, confusion, tension, and anger). Each item of the POMS is scored on a 5-point scale (0 means “Not at all” and 4 means “Extremely”). The total mood disturbance (TMD) score was calculated by the following formula: TM*D* = (Fatigue + Anger + Confusion + Tension + Depression) – positive mood (Vigor) + 100. A number of 100 was added to the total score to avoid negative numbers. In this investigation, Cronbach's α ranged from 0.86 to 0.93.

### Physiological measures

The maximum HR (HRmax) was estimated by carrying out the YIRT-1. The HR was measured via portable HR sensors (Polar Team Sports System, Polar-Electro OY, Kempele, Finland) at every 5-s interval during the RAS-E and RAS-NE. The HR peak is considered the highest value of HR attained in exercise training. The HR mean for each exercise modality (i.e., RAS-E and RAS-NE) was obtained. The %HRmax for each RAS trial was obtained [%HRmax = (HR mean/HRmax) × 100] (Květon et al., 2020).

Blood samples were taken at the fingertip of the index after each RAS intervention (i.e., RAS-E and RAS-NE), and the [La] was determined using a Lactate Pro Analyzer (Arkray, Tokyo, Japan) (Pyne et al., 2000).

### Statistical procedures

Data are presented as mean ± standard deviation. The assumption of normality was checked using the Kolmogorov–Smirnov test. The comparison between RAS-E and RAS-NE for the PACES score, the OMNI-RPE score, the POMS scores, and the physiological aspects measured were determined using Student's paired *t*-tests. To evaluate the differences, the effect size was calculated (Hopkins et al., 2009) and was determined as follows: <0.2 = trivial, 0.2–0.6 = small, >0.6–1.2 = moderate, >1.2–2.0 = large, >2.0–4.0 = very large, and >4.0, almost perfect. A two-way analysis of variance (ANOVA) was used to evaluate the effect of “exercise modality” (RAS-E and RAS-NE), “effort” (pre- and post-RSA), and “interaction” (exercise modality × effort) on mood responses (POMS scores). The 95% confidence level (*p* ≤ 0.05) is considered a significant result.

## Results

### Physiological responses

Physiological aspects presented in Table 1 indicate significant changes in HR mean, HR peak, [La], and RPE variables between children who received VE (RAS-E) and children who did not (RAS-NE). These values were higher in RAS-E than in RAS-NE.

**Table 1:** Results of exercise intensity between children who received VE (RAS-E) and children who did not (RAS-NE).

**Measurements**	**RAS-E**	**RAS-NE**	**|d|**	**Evaluation of the differences**
%HRmax (beat.min^−1^)	92.80 ± 1.31	93.40 ± 1.49^*^	0.37	Small
HR mean (beat.min^−1^)	173.35 ± 5.25	170.29 ± 6.03^*^	0.45	Small
HR peak (beat.min^−1^)	186.70 ± 6.54	182.35 ± 6.57^**^	0.66	Moderate
Lactate (mmol.l^−1^)	12.76 ± 2.25	11.47 ± 2.48^***^	0.56	Small
OMNI-RPE	7.52 ± 1.01	6.76 ± 1.25^**^	0.67	Moderate

^*^P < 0.05, ^**^P < 0.01, ^***^P < 0.001.

### Physical enjoyment

The perceived enjoyment score, calculated after exercise, is significantly higher (P < 0.0001, d = 2.8, very large) in the RAS-E (41.76 ± 2.90), compared to the RAS-NE (50.00 ± 2.96).

### POMS scores

**Figure 3:**
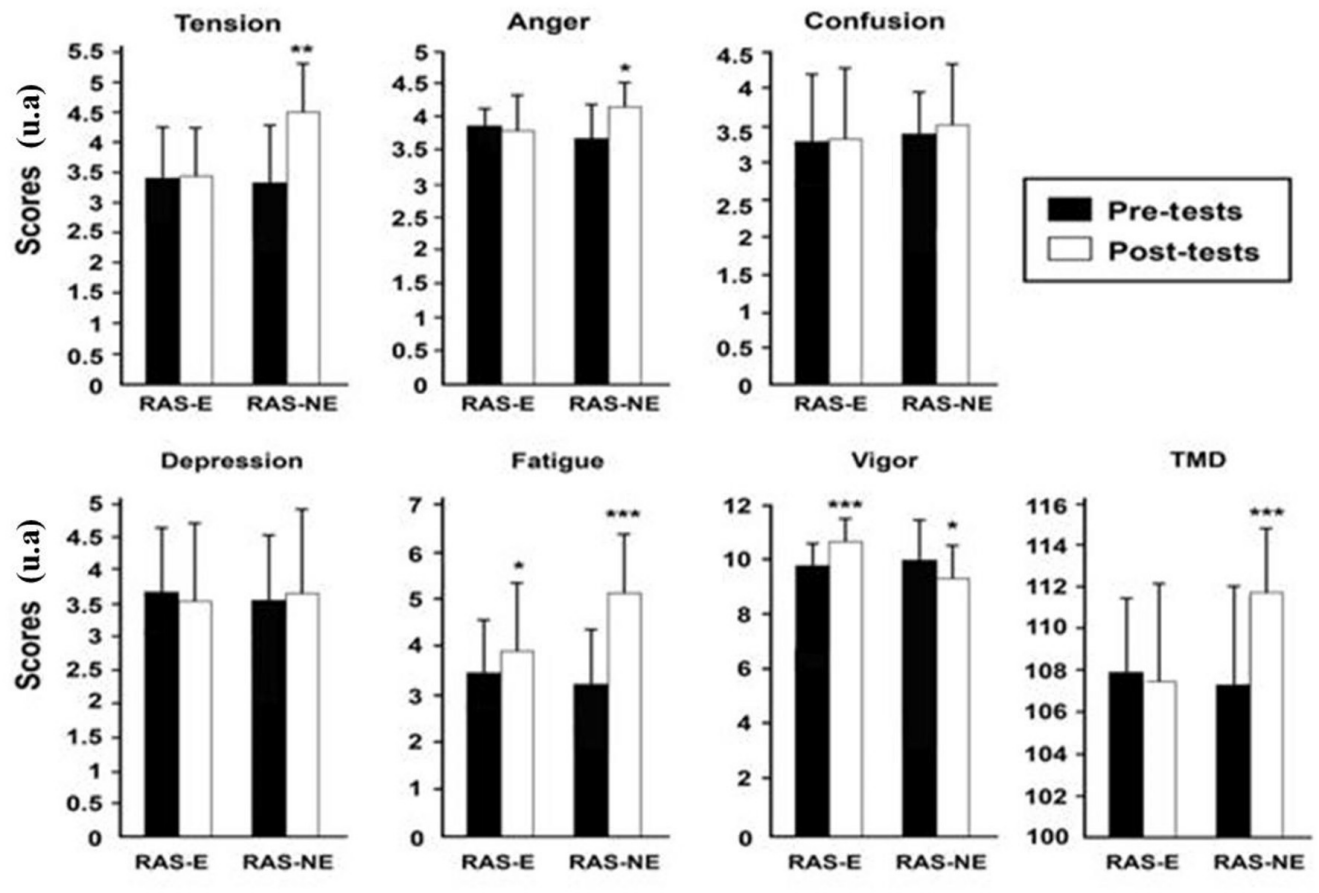
POMS scores for both repeated agility speed (RAS-E) and (RAS-NE) were measured before and after each intervention. TMD, total mood disturbance. *Significant difference between before and after RAS-E and RAS-NE. **p < 0.01, ***p < 0.001.

No significance of the main effects of exercise modality, effort, and interaction on confusion, depression, and anger scores was found. These emotional states were not significantly altered by VE. While there was a significant main effect of VE on TMD, tension fatigue, and vigor (Table 2, Figure 3).

**Table 2:** Mood state scores measured before and after RAS with VE and RAS without VE.

**Variables**	**Main effect of the exercise modality**		**Main effect of the effort**		**Interaction effect**	
	**F (1, 16)**	η^2^	**F (1, 16)**	η^2^	**F (1, 16)**	η^2^
Tension (u.a.)	0.1053	0.001	9.302^**^	0.088	7.831^*^	0.131
Anger (u.a.)	0.08602	0.004	3.303	0.015	1.215	0.033
Confusion (u.a.)	0.1356	0.002	1.00	0.005	0.4586	0.002
Depression (u.a.)	0.4848	0.002	0.6531	0.002	1.00	0.002
Fatigue (u.a.)	7.314^*^	0.007	48.94^***^	0.179	19.71^***^	0.303
Vigor (u.a.)	9.011^**^	0.004	5.728^*^	0.246	33.67^***^	0.061
TMD (u.a.)	0.49	0.002	54.23^***^	0.095	46.44^***^	0.105

TMD, total disturbance of mood.

^*^P < 0.05, ^**^P < 0.01, ^***^P < 0.001.

## Discussion

This investigation aimed to examine the influence of the coach's VE on the internal intensity and affective responses of youth soccer players during RAS training. The results showed that: (1) RAS-E increased HR peak, HR mean, %HRmax, and OMNI-RPE to a greater level than that of RAS-NE; (2) the PACES score was higher after RAS-E; and (3) RAS-NE produced a negative mood compared to that of RAS-E.

Regarding psychophysiological responses, this investigation indicated that the physiological responses and internal intensity were significantly higher during the RAS-E. This suggests that the children realized the training with intense effort, resulting in a high solicitation of the physical requirements (Kilit et al., 2019; Hammami et al., 2021). The encouragement variable increases HR peak, HR mean, %HRmax, [La], and OMNI-RPE. These results recommend that the coach's VE can encourage children to train at their maximum effort with a high work rate and a high level of commitment to engage in exercise training. The outcome of the present investigation is consistent with scientific studies that have reported that a coach's VE increases psychophysiological aspects and internal intensity in soccer players (Rampinini et al., 2007; Brandes and Elvers, 2017; Selmi et al., 2017b; Sahli et al., 2020; Aydi et al., 2022). For example, Selmi et al. (Lewis et al., 2011) mentioned the effectiveness and importance of VE in enhancing effort intensity during SSG (Selmi et al., 2017b). This result agrees with Aydi et al. (2022), who studied the impacts of VE on psychophysiological aspects during a physical-technical training circuit in young students. They showed that internal intensity and HR values were higher in physical activity with VE compared to physical activity without VE (Aydi et al., 2022). Similarly, Sahli et al. (Lewis et al., 2011) determined the impacts of VE on the game intensity during SSG in adolescent students. They suggested that RPE and HR variables were greater in SSG with VE than in SSG without VE (Sahli et al., 2020). Additionally, Rampinini et al. (2007) studied the impact of VE on internal intensity and physiological variables in soccer-specific training. They showed that HR, internal intensity, and [La] were higher during training with VE when compared to training without VE (Rampinini et al., 2007). These results suggest that the psychophysiological responses derived by RAS-E might vary according to the children's motivation, which a coach or physical coach can influence. Furthermore, these findings indicated that the VE factor was readily motivating for young players and could, therefore, more effectively improve physiological responses and engagement during training exercises.

Regarding physical enjoyment, recent studies increasingly report the use of the PACES, which measures the perceived physical enjoyment found in the practice of a given activity (Kilit and Arslan, 2018; Selmi et al., 2018; Kilit et al., 2019). In this investigation, we noticed that the perceived enjoyment score measured after the RAS-E was greater than that slowed after the RAS-NE. Our results suggest that VE has beneficial effects on the physical enjoyment of children during RAS training, conformant with recent studies that reported that athletes synchronized with VE had higher physical enjoyment (Selmi et al., 2017b; Kilit et al., 2019; Sahli et al., 2020, 2022; Aydi et al., 2022). Navarro-Paton et al. (Lewis et al., 2011) reported that increased physical enjoyment during physical activities may be associated with increased energy and improved cognitive performance (Navarro-Patón et al., 2019). For example, Aydi et al. (2022) confirmed that the PACES score measured after a soccer dribbling circuit exercise with VE was greater than that measured after a soccer dribbling circuit exercise without VE. Moreover, Sahli et al. (2020) indicated that SSG with VE led to a higher PACES score than SSG without VE. Indeed, VE during RAS training is associated with favorable emotional responses to physical activities and constitutes one of the important factors motivating young athletes to participate in physical exercises. We believe that the youth players in this investigation were motivated by the coach's VE and exhibited more positive behavior, as reflected by the high PACES scores. These results suggested that the perceived enjoyment derived from the physical activities may vary according to the modality of the training activity, the coach's communication, the results, and the motivation of the participants (Selmi et al., 2017b; Kilit and Arslan, 2018; Aydi et al., 2022). Additionally, these findings suggested that the VE was significantly encouraging and agreeable for the children and could thus be an important strategy for enhancing positive emotional responses during training.

The mood state during training is an important characteristic of performance (Hashim et al., 2011; Selmi et al., 2018; Sparkes et al., 2018). The POMS is frequently used to assess the affective status of athletes during physical activity (Hashim et al., 2011; Selmi et al., 2020). This investigation indicated a significant increase in the fatigue score in both exercise modalities (i.e., RAS-E and RAS-NE), suggesting that both exercise modalities induced a similar perception of tiredness. These findings agree with a recent study (Lewis et al., 2011) that examined the effects of high-intensity interval training (HIIT) and SSG on the mood state of soccer players (Selmi et al., 2017a). They indicated that both training methods significantly increased the fatigue scores measured by the POMS questionnaire.

RAS-NE induced a significant increase in TMD, unlike RAS-NE, which resulted in no changes. Performing the RAS-NE causes not only an increase in fatigue as seen in the RAS-E but also a significant decrease in vigor and a significant increase in anger and tension, suggesting that during unmotivated exercises, participants commonly reported negative variations in their POMS score (Lewis et al., 2011). These results are reliable with several investigations that have indicated that VE is an important factor of positive feelings in athletes (Selmi et al., 2017b; Sahli et al., 2020; Aydi et al., 2022). These results align with the investigated impact of the coach's VE on mood state in adolescent male students during a physical-technical exercise (Aydi et al., 2022). They showed that the circuit exercise with VE resulted in a significant improvement in mood. Thus, Sahli et al. (Aydi et al., 2022) showed that soccer games (4 vs. 4) with VE produce a positive mood state compared to that of the games without VE, indicating TMD, fatigue, and anxiety increased and positive mood decreased after games without VE in adolescent players (Sahli et al., 2020). The results demonstrated that the coach's behavior and encouragement positively influenced the mood state during RAS training among youth soccer players. It has been suggested that the negative mood states during RAS-NE are related to insufficient motivation and a lack of pleasure, which are linked with unpleasant emotional perceptions (Selmi et al., 2018; Aydi et al., 2022). These results suggest that the motivation factor during training causes positive emotion in youth soccer players, unlike unmotivated activities (Lewis et al., 2011).

### Limitations

Collectively, the present investigation suggests that coach-provided VE may produce high motivation in children, producing improvements in their physical enjoyment, mood state, and physiological responses. This study revealed that RAS-E was characterized by an increased PACES score and positive mood and induced higher exercise intensity among children. While these results are interesting to highlight the positive responses to RAS exercise in young soccer players, there were some limitations. The number of participants was limited due to the complexity of recruiting many homogeneous players. The study integrated only male players in the U-14 division. Only one form of RAS training and only one age category were used in this investigation. Finally, comparing these aspects with the performance RAS measurements (i.e., peak time, total time, fatigue index, and average time) would be interesting.

### Conclusion

The present investigation shows that the internal intensity, the physiological responses (i.e., HR peak, HR mean, HR), the perceived positive mood, and the physical enjoyment are superior during RAS-E compared to RAS-NE. VE is suggested to be an important and effective method to improve training intensity, mood state, and physical enjoyment during soccer-specific exercises with high intensity. The findings imply that coaches should verbally encourage players more often in order to increase their motivation, physical engagement, and involvement during physical training.

### Practical applications

This investigation offered practical implications. To the best of our knowledge, this is the first investigation to examine exercise intensity and affective aspects during RAS training in young soccer players. The VE of coaches can be seen as an essential factor during an intense exercise, as it elicits an important physiological solicitation and a positive psychological state. For this reason, coaches must verbally encourage their young players during intensified physical activities (i.e., RAS) to improve the exercise intensity, enhance players' physiological aspects, and create a positive feeling.

## Data availability statement

The raw data supporting the conclusions of this article will be made available by the authors, without undue reservation.

## Ethics statement

The study was conducted in accordance with the Declaration of Helsinki and the protocol was approved by the local research Ethics Committee of the High Institute of Sport and Physical Education of Kef, Tunisia. Written informed consent was obtained from each participant's parents/legal guardians after being thoroughly informed about the purpose and potential risks of participating in the study. Written informed consent to participate in this study was provided by the participants' legal guardian/next of kin.

## Author contributions

All authors listed have made a substantial, direct, and intellectual contribution to the work and approved it for publication.
